# Size Effects in Single- and Few-Layer MoS_2_ Nanoflakes: Impact on Raman Phonons and Photoluminescence

**DOI:** 10.3390/nano12081330

**Published:** 2022-04-12

**Authors:** Sandra Cortijo-Campos, Carlos Prieto, Alicia De Andrés

**Affiliations:** Instituto de Ciencia de Materiales de Madrid, CSIC, C/Sor Juana Inés de la Cruz, Cantoblanco, 28049 Madrid, Spain; s.cortijo@csic.es (S.C.-C.); cprieto@icmm.csic.es (C.P.)

**Keywords:** nanoflakes, 2D-materials, Raman, photoluminescence, defects, excitons

## Abstract

The high optical absorption and emission of bidimensional MoS_2_ are fundamental properties for optoelectronic and biodetection applications and the opportunity to retain these properties in high quality nano-sized flakes would bring further possibilities. Here, a large set of single-layer and few-layer (2–3 layers) MoS_2_ flakes with size in the range from 10 nm to 20 μm are obtained on sapphire by vapor deposition techniques and evaluated combining the information from the Raman phonons with photoluminescence (PL) and absorption bands. The flakes have triangular shape and are found to be progressively relaxed from the tensile strain imposed by the sapphire substrate as their size is reduced. An increasing hole doping as size decreases is deduced from the blue shift of the A_1g_ phonon, related to charge transfer from adsorbed oxygen. No clear correlation is observed between defects density and size, therefore, doping would be favored by the preferential adsorption of oxygen at the edges of the flakes, being progressively more important as the edge/surface ratio is incremented. This hole doping also produces a shift of the PL band to higher energies, up to 60 meV. The PL intensity is not found to be correlated to the size but to the presence of defects. The trends with size for single-layer and for 2–3 layer samples are found to be similar and the synthesis method does not influence PL efficiency which remains high down to 40 nm being thus promising for nanoscale photonics.

## 1. Introduction

MoS_2_ is one of the most studied bidimensional TMDCs because of its excellent light absorption and emission properties. The electronic structure of bulk 2H-MoS_2_ is modified as the number of layers is reduced and, from few- to single-layers, a transition from indirect to direct bandgap occurs for the bidimensional material [1] so that PL quantum yield increases and an emission band, around 680 nm, becomes detectable for few-layer and is maximum for single-layer MoS_2_ [2] thus, obtaining single-layer samples is thus crucial for most applications. The PL emission band is complex, it is in general dominated by the A exciton at ~1.83 eV (680 nm). The weaker B exciton, at ~2 eV, is detected in the high energy side of A component and, for n-doped samples, a trion band becomes significant. 2D-MoS_2_ has already shown excellent performance in a wide variety of applications related to its optical characteristics, from photodetectors [3,4] to biosensors [5,6].

The design and fabrication of quantum dots, nanodots, nanosheets or nanoflakes of 2D materials, initially of graphene and more recently of transition metal dichalcogenides (TMDCs), is a topic of increasing interest due to the new possibilities that these materials offer for applications. In the very small lateral size limit, 1D-MoS_2_ nanodots or nanoflakes, a wide visible emission band is observed independently of the number of layers. It is originated by modifications of the electronic structure due to quantum confinement giving rise to direct interband transitions. The size-tunable visible photoluminescence as well as the excitation dependent PL confer further potential for applications. Initially, MoS_2_ nanoflakes were obtained by liquid exfoliation for a variety of applications [7,8,9,10] with, however, broad distributions of the in-plane size (from few to thousand nm) and number of layers. Moreover, solvent remains and layer aggregation are disadvantages. The reported nanodots present a broad emission in the visible with a wavelength that is dependent on the size and on the excitation energy [11,12,13]. However, broad blue PL (~415 nm) was reported to be independent of the excitation for single-layer nanosheets with uniform size (~16 nm) [14]. Using top-down approaches, few and single-layer nanoflakes, with controlled sizes in the range from ~30 to 80 nm diameter and very narrow size distributions, can be obtained by patterning CVD or mechanically exfoliated flakes [15].

The origin of the PL wavelength dependence on the excitation energy has been sometimes related to the wide size distribution of the nanodots [13] and its dependence with size is argued to be related to quantum confinement. However, the role, in the PL characteristics, of size, size distribution, number of layers and defects are still not well established and, the limits for quantum confinement, as whether this effect is important for sizes >10 nm, are still unclear [12,15]. Moreover, experimentally, the wavelength shows an unclear dependence with size. For example, the emission was reported to vary from 480 to 500 nm for 2 to 26 nm diameters [16] but a much larger variation (430–610 nm) has been published for narrower particle size range (2–7 nm) [17]. Increased photoluminescence (PL) wavelength by quantum confinement is expected for sizes close to the exciton Bohr radius, *r*_B_, that is estimated to be around 2 nm for MoS_2_ [15,18]. However, a weak quantum confinement regime, for sizes >> *r*_B_, is also proposed to explain the PL energy blue shift observed for 30–80 nm flakes. The shift, up to around 24 meV, depends on size and on whether the measurements are done in air or in vacuum [15]. The PL blueshift of round shaped 2D-quantum dots directly grown on silicon by CVD with ~40 nm diameter and 3 layers was also assigned to weak confinement and a 6 fold increased intensity is reported [19].

On the other hand, the emission band of 2D-transition metal dichalcogenides is very sensitive to several different parameters such as ion intercalation, interaction with the substrate or other layers, or to doping. For example, the deposition of InGaN quantum dots on WS_2_ single layers has been shown to induce quasi-0D photon emission of the WS2 [20]. It is well known that the PL in 2D-MoS_2_ is very sensitive to doping. In particular, p-doping, blue shifts the PL emission energy and increases its intensity [21,22]. The p-doping is often related to hole transfer from oxygen adsorbed molecules to the 2D-MoS_2_; indeed, a significantly larger PL energy increase in ambient atmosphere than in vacuum is reported as the flake size is reduced.

Reducing the size of 2D-materials is an objective but also a challenge since the control required to obtain real single-layer and high quality nano-sized flakes is not straightforward. A detailed analysis of the PL behaviour with size combined with an adequate characterization of the number of layers, strain and defects are required to identify the underlying mechanisms. The careful characterization that nano-sized single-layer materials require can be compromised since standard well established procedures may not rule. As an example, the most commonly used parameter for the direct estimate of the number of layers, *N*, is the frequency difference (Δ*ω*) between the A_1g_ and E_2g_ first order Raman phonons, Δ*ω* = A_1g_ − E_2g_ [23,24]. However, samples obtained by different vapour deposition techniques, can present strain, doping, defects and ionic substitution or adsorption that modify the Raman characteristics, as briefly summarized in the next section. In these cases, Δ*ω* may be inadequate for the estimation of the number of layers.

In this work we study the modifications of MoS_2_ single and few-layer (2–3) flakes with size in the range from 10 nm to few micron, combining the analysis of the Raman characteristics with those of PL and of reflectance/transmittance spectra. The combination of these techniques allows discerning between the characteristics in the Raman spectra originated by different types of defects or structural distortions with that related to the number of layers. We show that the blue shift (up to ~50 meV) observed in the PL band as size is reduced is due to p-doping, both for the single-layer and the 2–3 layer nanoflakes, as indicated by the A_1g_ frequency modifications. The strain, usually imposed by the substrate in CVD growth, is progressively relaxed as size decreases. Interestingly, no clear correlation is detected between defect concentration and reducing size. For flakes with sizes much larger than Bohr radius, doping is probably more relevant than confinement effects.

### Raman Spectroscopy for the Characterization of 2D-MoS_2_

The point groups of hexagonal MoS_2_ with even *N* (D_3h_), odd *N* (D_3d_) and bulk (D_6h_) are different and so are the irreducible representations of their vibration modes [25]. Here, for simplicity, we will use those of bulk (D_6h_). The correspondence between the irreducible representations for 1L (D_3h_)–2L (D_3d_)–bulk (D_6h_) being: A’–A_g_–A_1g_, E’–E_g_–E_2g_, E’’–E_g_–E_1g_ [26].

**Figure 1:**
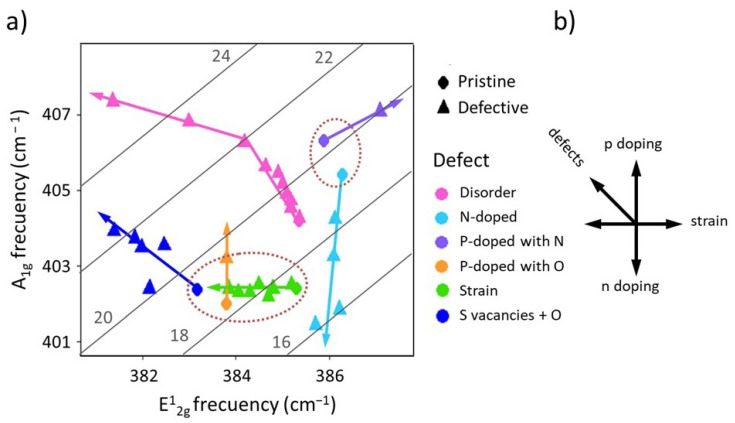
(**a**) Variation of A_1g_ versus E_2g_ Raman frequencies for 1L 2D-MoS_2_ collected from literature [28,35,37,39,42] upon n or p doping, strain, atomic substitution or adsorption, or disorder (defects induced by ion bombardment). The diagonal lines correspond to constant Δ*ω* (the values are indicated in the figure). (**b**) schema of the general behaviour of these phonons. The figures were created from the data in literature.

It has been reported that E_2g_ frequency increases under compressive strain (either uniaxial or biaxial) while A_1g_ remains constant, thus, Δ*ω* increases with strain [27,28,29,30,31]. Phonon confinement is promoted by ion bombardment by generating monoatomic or polyatomic vacancies that, due to the slope of the dispersion relations around Γ, produces an increase of A_1g_ and decrease of E_2g_ frequencies so, again, increasing Δ*ω* [32,33,34,35,36]. Doping, without involving ionic substitution, modifies only A_1g_ frequency, increasing or decreasing Δ*ω* for p and n doping, respectively. The creation of S vacancies combined with oxygen adsorption produces lattice distortions and also p doping so that Δ*ω* increases while *N* substitution increases both A_1g_ and E_2g_ frequencies [37,38,39,40,41,42]. Figure 1 collects A_1g_ and E_2g_ frequencies from published experiments on large single-layer MoS_2_ obtained either by mechanical exfoliation (ME) or by chemical vapour deposition (CVD) exposed to uniaxial and biaxial strain, n and p doping, ion bombardment for the generation of defects or adsorption of O_2_. The arrows indicate the direction of the variation of the phonon frequencies and the diagonal lines correspond to constant frequency difference, Δ*ω*, illustrating the wide variation of this parameter for single-layer MoS_2_: Δ*ω* varies from ~18 to ~24 cm^−1^. Note that the initial 1L-MoS_2_ samples show large variability of A_1g_, E_2g_ and Δ*ω* values.

To summarize, Δ*ω* increases by the creation of point defects or when p doping and lattice distortions are combined. On the contrary, Δ*ω* decreases for tensile uniaxial or biaxial strain or for electron doping. The variations of Δ*ω* due to these effects are sufficiently large (>4 cm^−1^) so that the identification of 1L, 2L and 3L films can be erroneous when using the standard behaviour of Δ*ω* vs. *N* for ME or pristine CVD samples. For ME samples, single layers are characterized by Δ*ω* = 19–20 cm^−1^ and Δ*ω* increases up to around 25 cm^−1^ for more than 4 or 5 layers [23,24].

## 2. Materials and Methods

### 2.1. MoS_2_ Nanoflakes and Reference Samples

The nanoflakes were grown using two vapor techniques: chemical vapor deposition (CVD) and physical vapor transport (PVT). Samples grown by PVT and by mechanical exfoliation with large flakes (>20 μm) were used as references.

CVD growth process is based on a gas-phase reaction between MoO_3_ (99.99% purity, Alfa Aesar Kandel, Germany) and sulfur evaporated from solid phase (99.999% purity, Alfa Aesar Kandel, Germany). The MoO_3_ powder, 14 mg, was loaded into a quartz tube placed in a furnace (Ceramic fiber heater, Watlow Electric Manufacturing Co., Saint Louis, MO, USA) together with the sapphire (0001) substrates, both at ~680 °C. A crucible, located upstream from the substrates, at 200 °C, contained 1 g of sulfur. Ultrahigh-purity Ar was used as the carrier gas. Different pressure values (10^−2^–2 mbar) and Ar flux (10–100 sccm) were used. The substrates were either directly put at the bottom of the quartz tube or located on a graphite holder forming a 45° angle with the Ar flux. Growth temperature was maintained for 20 min and then cooled down to room temperature. The resulting flakes presented sizes in the 10–500 nm range depending mainly on the positioning of the substrate in the Ar stream. PVT samples were grown in a quartz tube furnace (Ceramic fiber heater, Watlow Electric Manufacturing Co., Saint Louis, MO, USA) using a single precursor source consisting in MoS_2_ powder and sapphire (0001) as substrates [43]. The precursor is placed at the center of the tube at 970 °C and the substrate is placed downstream, at approximately 820 °C. The growth was performed at a pressure of 1 mbar under 20 sccm Ar flow, maintaining the growth temperature constant for 20 min. The resulting flakes presented sizes in the 1–5 µm range. The PVT reference large flakes were obtained in similar conditions with a pressure of 10 mbar.

### 2.2. Characterization Techniques

The size and morphology of the nanoflakes were analyzed using atomic force microscopy (AFM AFM, Cervantes Fullmode Atomic Force Microscopy System, Nanotec, Madrid, Spain) in the tapping mode (equipment and software from Nanotec^TM^). Commercial tips (Nanosensors PPP-EFM-50, Nanosensors, Neuchatel, Switzerland) with force constant 0.5–0.95 N/m and *f*_0_ ≈ 45–115 kHz were used. Micro-Raman and micro-PL experiments were performed at room temperature using the 488 nm line of an Ar^+^ laser (3 mW) (Stellar-Pro, Modu-Laser, South Centerville, UT, USA). An Olympus (Tokyo, Japan) microscope with a x100 objective with a high optical aperture (N.A. = 0.95) allows for <0.8 µm lateral resolution. The scattered light was filtered with a notch filter (Kaiser) (Greenwood, IN, USA) and analyzed with a Horiba (iHR-320) (Kioto, Japan) monochromator (1800 L/mm grating) coupled to a Peltier cooled Synapse CCD (Kioto. Japan). To compare Raman intensities obtained from different samples, all spectra are corrected using the Raman signal recorded for silicon phonon at 520 cm^−1^. Micro-reflectance experiments were done using a white light lamp, an Olympus microscope (×100 objective) (Kioto. Japan) and a spectrograph with a 150 L/mm grating, coupled to a Peltier cooled CCD (both from CVI Laser corporation, Alburquerque, NM, USA). A 500 nm pinhole is used for spatial filtering. The reflectance of the substrate at a position close to that of the flake is measured and used to obtain the reflectance of the films: *R*(2D-MoS_2_) = *R*_measured_/*R*_substrate_.

## 3. Results and Discussion

**Figure 2:**
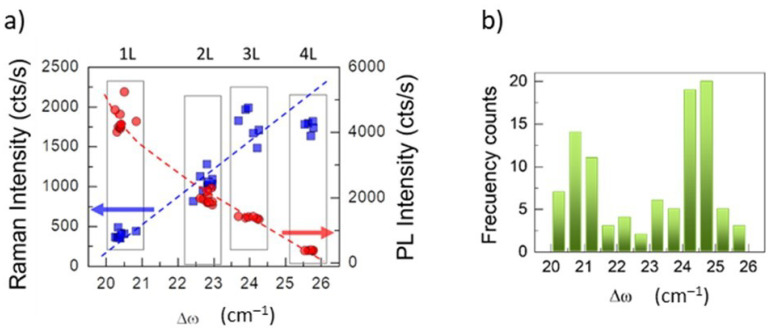
(**a**) standard behaviour of Raman A_1g_ and PL intensities for PVT large flakes (20 µm) versus Δ*ω* which is directly related to the number of layers; (**b**) statistics of Δ*ω* for several nanoflakes.

The standard behaviour of the intensities of Raman A_1g_ peak and PL band for large (tens of microns) MoS_2_ flakes as the number of layers increases is shown in Figure 2a. The data correspond to the reference samples that consist in large flakes (~20 μm) obtained by PVT. The Intensities are plotted vs. Δ*ω* and 1L, 2L, 3L and few-layer flakes can be easily identified (blue squares). Typically, Δ*ω* = 19–20 cm^−1^ corresponds to single-layers while Δ*ω* = 21.5–22.5 cm^−1^ corresponds to double-layers. The intensity of A_1g_ phonon increases linearly for one to three layers while PL intensity (red dots) is rapidly quenched with *N*. However, for MoS_2_ nanoflakes with different sizes, obtained by CVD and PVT, the situation is more complex and the determination of the number of layers using Δ*ω* is not straightforward. Figure 2b shows the statistics of Δ*ω* values obtained from many nanoflakes. In these cases, the boundary between the flakes with 1 and 2 layers is unclear. The first maximum corresponds to 1L and the second one, probably to 2 and 3 layers.

**Figure 3:**
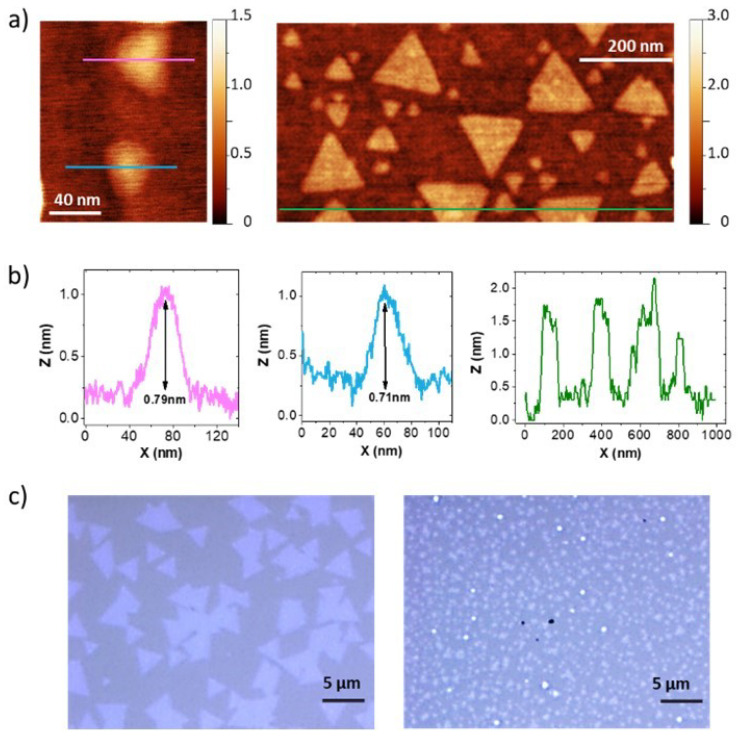
(**a**) Topographic AFM images of two MoS_2_ samples with nano-flakes grown by CVD on sapphire; (**b**) profiles along the lines indicated in the AFM images showing the size and height of the nanoflakes; (**c**) optical images of two MoS_2_ samples with flakes ~4 µm and ~1 µm grown by PVT on sapphire.

The smallest MoS_2_ flakes, 10–100 nm, (Figure 3a) were obtained by CVD from MoO_3_ and S powders either with high Ar flux (100 sccm) at moderate pressure (2 mbar) or with low flux (10 sccm) and low pressure (10^−2^ mbar) and with the substrate lying at the bottom of the quartz tube. In these conditions, the amount of deposited MoS_2_ on the substrates is reduced so that the flakes do not grow in size. In the first case, the high Ar flux and relatively low pressure evacuates rapidly the gas precursors. In the second case the low pressure acts in the same way. Larger flakes, ~500 nm, were obtained positioning the substrate in a graphite support forming an angle of 45° with the gas flux at 45 sccm and 1 mbar. The main parameter that controls the size of the flakes on sapphire substrates seems to be the velocity of extraction of the precursors (MoO_3_ and S) and thus the time allowed reacting and depositing on the substrate. The position of the substrate relative to the stream is the most determining parameter to obtain small flakes, obtaining the smallest flakes when the substrates are parallel to the flux and larger flakes when it forms a 45° angle. The combination of the Ar flux entering into the tube and pressure measured at its exit defines the velocity of the flux of the precursors.

The intermediate range flakes, 1–5 µm, were obtained by PVT (from MoS_2_ powder) at 20 sccm and 1 mbar, with the substrate also forming a 45° angle with the Ar flux. Figure 3a,b presents AFM images of samples with nanoflakes, obtained by CVD, as well as profiles showing that the height of the nanoflakes varies between ~0.75 to ~1–3 nm. Optical images of samples with micron-sized flakes obtained by PVT are shown in Figure 3c. The flakes have a triangular shape in all cases as well as the reference PVT samples with large flakes (tens of microns [44]).

Since Δ*ω* can be modified due to strain/defects/doping as explained previously, macroscopic and microscopic transmittance and reflectance spectra were recorded to find out whether the samples are single-layer or not. The modification of the electronic band structure with the number of layers, *N*, [45] is reflected in the transmittance and reflectance spectra that present three features corresponding to excitons A (~1.88 eV), B (~2.05 eV) and C (~2.6–2.8 eV). A and B excitons are due to transitions at the K point of the Brillouin zone while C is related to a band nesting condition [46] in the Γ-Q direction. Small changes occur in A and B excitons with *N*, the larger modifications of the band structure occur at the band nesting condition. Thus, the energy of C exciton varies importantly, from ~2.8 eV for single layer to ~2.6 eV for few layers. The change occurs almost totally from 1 to 2 layers, [26,45] the energy of C exciton is thus an excellent indicator of the single-layer character.

**Figure 4:**
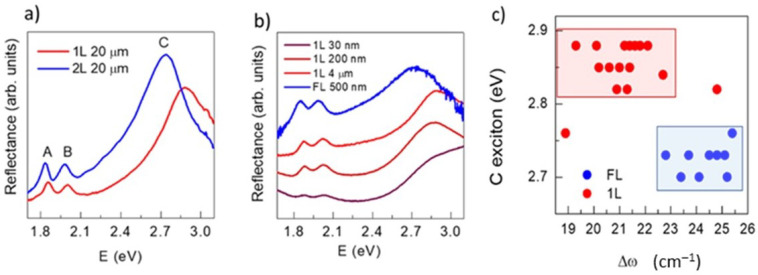
(**a**) micro-reflectance spectra of large MoS_2_ flakes (20 μm) with 1L and 2L; (**b**) macro or micro- reflectance spectra of samples with flakes of different sizes. The A, B and C excitons are indicated; (**c**) C excitation energy versus Δ*ω*. Red symbols correspond to single-layer and blue to few-layer flakes.

Differential reflectance (*R*(sample) − *R*(substrate))/*R*(substrate) = *R*(sample)/*R*(substrate) − 1) almost coincides with differential transmittance (*T* = 1 − *R*), for sufficiently thin samples, so these can be easily compared. Figure 4a presents reflectance divided by the substrate signal (*R*(sample)/*R*(substrate)) either obtained from micro-reflectance or from micro or macro- transmittance measurements. Figure 4a shows micro-reflectance spectra of a reference sample with large flakes (20 µm) with one and two layers in concordance with Raman and AFM. The C exciton is shifted by ~0.3 eV from 1 to 2 layers. Figure 4b shows the transmittance spectra of 1L samples with 30 nm, 200 nm and 3 µm flakes and that of a sample with 500 nm with 2–3 layers (FL).

Plotting the energy of the C exciton vs. Δ*ω* for the different samples (Figure 4c), evidences that Δ*ω* for single-layer nanoflakes varies from 20 to 23 cm^−1^ and that around 22–23 cm^−1^ samples may be either 1L or >1L, N being defined by the C exciton energy. Another excellent indicator of the single-layer character is the non-detection of the E_1g_ phonon that has been recently shown to be detectable only for flakes with more than 1 layer [26] (Figure S1 in Supplementary Materials). However, the Raman spectra must have a high signal/noise ratio to clearly detect the E_1g_ peak, which is not always easily reachable. Once the single-layer or few-layer character of the samples is defined, the characteristics of Raman and photoluminescence spectra can be properly analysed. In the subsequent figures, it is indicated whether the flakes are single-layer (red dots) or few-layer (blue dots) according to their C exciton energy. Data from the MoS_2_ reference samples with large flakes (20 µm) are included.

**Figure 5:**
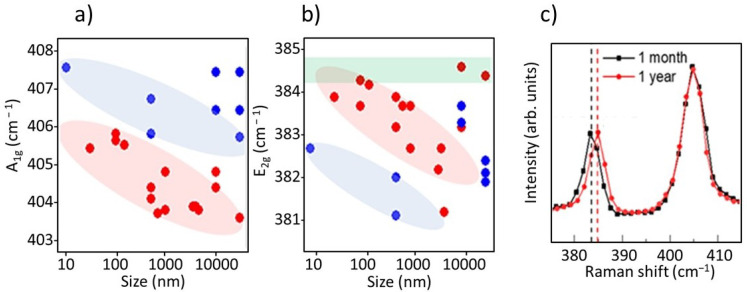
(**a**) A_1g_ and (**b**) E_2g_ frequencies as a function of the size of the flakes, red dots correspond to 1L and blue dots to few-layer samples. (**c**) Raman spectra of large flakes (20 µm), one month (black line) and one year (red line) after the sample growth.

Figure 5 shows interesting trends of the two main first order Raman phonons of MoS_2_ with the flake size. A_1g_ and E_2g_ frequencies increase as the size of the flakes is reduced while their difference, Δ*ω*, shows no correlation with size (not shown). The trends of single-layer (red area) and few-layer (blue area) are parallel but vertically shifted, upwards for A_1g_ and downwards for E_2g_, consistent with the sign of the frequency changes of these phonons with the number of layers.

E_2g_ frequency requires special attention. Mechanically exfoliated (ME) samples transferred on different substrates present E_2g_ frequencies within the green rectangle (>384 cm^−1^) (Figure 5b), similarly to large PVT flakes months after their growth. On the contrary, as grown large PVT flakes present a significantly lower frequency (around 383 cm^−1^) (Figure 5c). The as grown PVT large MoS_2_ flakes are stretched (tensile strain) by the sapphire substrate (or by the different expansion rates during the cooling process, [31]) and then slowly relax towards the unstrained ME value, many months after. The as-grown small flakes are also stretched, thus decreasing the standard E_2g_ frequency, but the impact of the substrate is reduced as the size of the flake decreases and, for the smallest flakes, the unstrained E_2g_ values (for 1L and FL) are observed, indicating relaxed flakes. The variation of strain is estimated to be around 2% according to [28]. On its side, A_1g_ frequency increases as size is reduced. This variation can be explained by an increasing p doping as size shrinks. Hole doping is typically due to charge transfer from adsorbed oxygen usually at sulphur vacancies [21,39,40,41].

**Figure 6:**
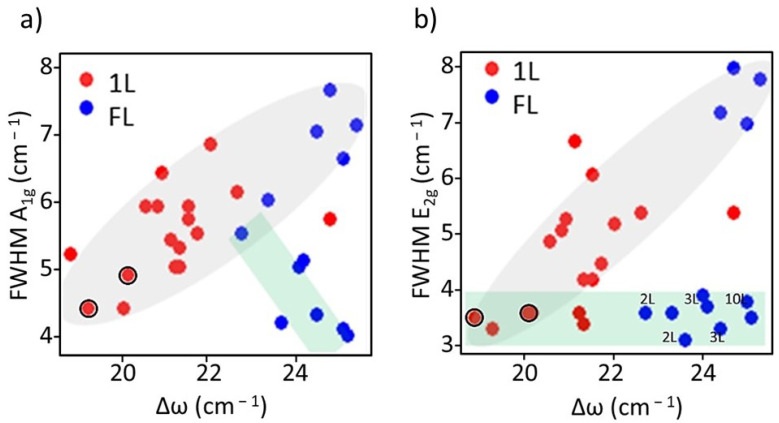
Dependence of the FWHM of (**a**) A_1g_ and (**b**) E_2g_ versus the frequency difference, Δ*ω* for nanoflakes (grey ellipses) and reference samples (green rectangles). The data of the 1L reference samples are indicated with black circles and the number of layers for most of the FL references flakes are indicated in the right panel.

Another characteristic of the Raman peaks that can give valuable information is their width. In the case of defect-free large 2D-MoS_2_ flakes, the FWHM of E_2g_ is independent of N while that of A_1g_ is slightly reduced as *N* increases. In this case, Δ*ω* is exclusively controlled by the number of layers. However, defects and disorder have been shown to increase the full widths at half maximum (FWHM) of A_1g_ and E_2g_ peaks, and, as previously indicated, Δ*ω* also increases [34,35]. In Figure 6 the FWHM of A_1g_ and E_2g_ peaks are plotted as a function of Δ*ω*. The data corresponding to the reference samples (large flakes) are inside the green rectangles. As expected, the FWHM of A_1g_ decreases for bigger Δ*ω* (=*N* increase) while that of E_2g_ is constant. Looking at the FWHM of the Raman peaks of the nanoflakes, Figure 6a,b, a clear correlation is observed with Δ*ω* for both peaks. Since in this case the number of layers and disorder influence Δ*ω*, it is necessary to look to the 1L and few-layer data separately. The correlation is clear for the red dots, that all are single-layer flakes. So Δ*ω* is a good parameter to assess the presence of disorder/defects in nanoflakes.

Maintaining high PL yield as the size is reduced is a requirement for optoelectronic applications of MoS_2_ nanoflakes so, analysing the behaviour of the PL intensity as size is reduced is essential. To reliably compare the PL efficiency of different samples we have used the intensity ratio between the PL band and the A_1g_ Raman peak, I(PL)/I(A_1g_); Raman signal is used to normalize and make the data comparable. This normalization is also required when looking at nano-sized flakes with different sizes and densities. The spot of the laser used for Raman and PL measurements, with the ×100 objective and 488 nm excitation, is ~0.8 µm in diameter so the signal is originated by a variable number of flakes and a variable fraction of MoS_2_ area within the laser spot.

**Figure 7:**
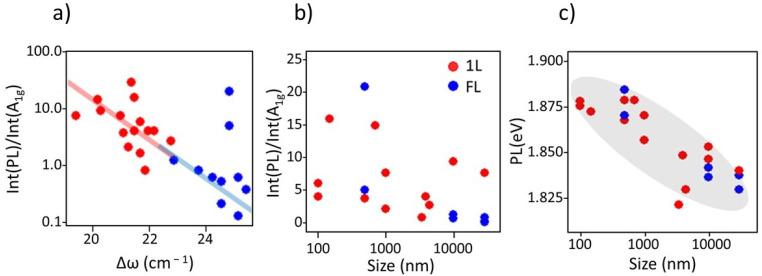
Ratio of PL and A_1g_ intensities versus (**a**) the frequency difference, Δ*ω*, and (**b**) the size of the flakes; (**c**) the position of the PL band in eV versus the flake size.

The dependence of the PL intensity (I(PL)/I(A_1g_) ratio) with the frequency difference, Δ*ω*, is shown in Figure 7a and its behaviour with the flake size in Figure 7b. No clear correlation is found between I(PL)/I(A_1g_) and size (Figure 7b) but the PL efficiency is drastically reduced with Δ*ω* (Figure 7a). An exponential dependence (note the log scale for the intensity ratio) is observed in the whole Δ*ω* range, from 19 to 25 cm^−1^, that includes single-layer (red dots and red line) and few-layer samples (blue dots and blue line). Δ*ω* varies with the number of layers due to the effect of the weak but non-zero interlayer coupling and the long range screening that modifies the of the A_1g_ and E_2g_ frequencies as adjacent layers are included, this is an intrinsic effect. The modifications of the phonon frequencies by external means (strain, doping, size, defects) can thus be called extrinsic. In the single-layer samples, only extrinsic effects are operating while, for multi-layers, both may have a role, however, in the case of the reference samples, intrinsic one is dominating. Figure 7a indicates that increasing Δ*ω* depletes PL intensity independently of the origin of the changes in Δ*ω* in spite of the different processes occurring (increased disorder and N increase).

Doping has a strong impact on the position and intensity of the PL band in MoS_2_ which, at room temperature, is mainly originated by the recombination of A and B excitons and a negative trion (X^−^). Looking at the energy of the PL band, a clear blue shift, up to 50 meV, is observed as the size is reduced (Figure 7c). This blue shift is consistent with the increased p-doping previously deduced from the A_1g_ frequency trend (Figure 5a) as size shrinks. Hole doping usually enhances PL intensity and increases its energy, but the involved mechanisms are still not totally clear. Calculations foresee the formation of mid-gap localized levels due to sulphur vacancies, often present in as grown samples, that are can be repaired by O_2_ adsorption [47,48] increasing absorption and largely shifting the exciton energies. Alternatively, the large observed increase of PL band intensity upon hole doping has also been assigned to the depletion of the trion X^−^ recombination pathway in favour of exciton processes [21].

**Figure 8:**
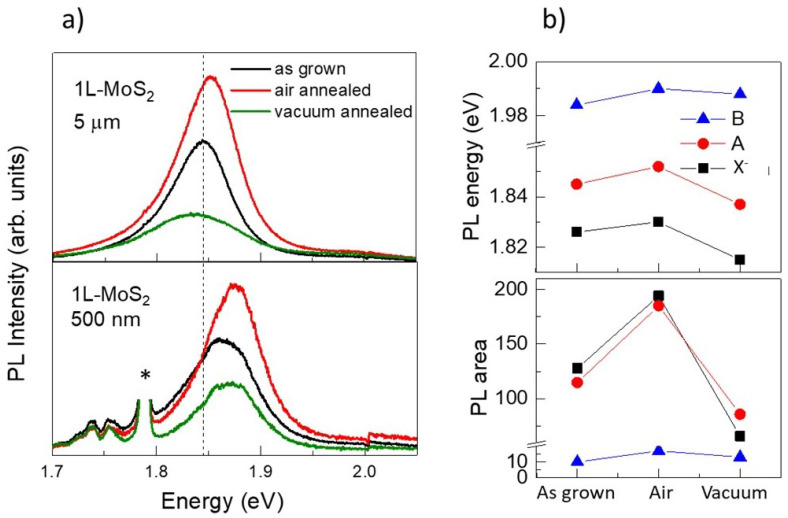
(**a**) PL spectra al RT of MoS_2_ single-layer samples with 5 µm and 500 nm flakes, as grown (black lines), after an annealing in air (red) and then an annealing in vacuum (green). Excitation is 488 nm. The asterisk indicates the emission peaks of Cr impurities in the sapphire substrate. (**b**) energies and intensities of the A and B excitons and the negative trion from the fits to the PL of a 5 µm flake as grown and after two successive annealing treatments, one in air and the second in vacuum.

To further assess the relevance of oxygen, two samples, with 5 µm and 500 nm flakes, were first annealed in air at 300 °C for 30 min, to favour O_2_ adsorption onto the MoS_2_ surface. Afterwards, the samples were annealed, also at 300 °C, in vacuum (10^−5^ mbar) for one hour, to desorb O_2_. Figure 8a shows clearly the blue shift of the PL band from the larger to the smaller flakes (top and bottom figures). A smaller blue-shift is also detected after the air annealing as well as an increase of the PL intensity (red lines), related to the healing of the defects by the O_2_ physical or chemical adsorption. The PL bands for the 5 µm sample have been fitted to 3 Gaussian functions corresponding to A and B excitons and to the X^−^ trion (Figure S2 in Supplementary Materials). The results from the fits of the positions and peak areas are shown in Figure 8b. The PL bands were previously normalized to the Raman intensity. The red-shift of the vacuum annealed flakes is evident. This last treatment partly eliminates adsorbed oxygen thus reducing the p-doping of the sample but also produces defects in the lattice that provides extra non-radiative recombination pathways. These observations are consistent with the proposed hole transfer from adsorbed O_2_ to the MoS_2_ nanoflakes upon size reduction for the modification of the PL energy.

Looking at the behaviour of the intensities of the three components of the PL band in Figure 8b, the reported possible transfer between the X^−^ to the A and B excitons optical relaxation processes when p-doping (air annealed vs. as grown) is not evident. The areas of the A and trion components (the fits are shown in Figure S2) show very parallel behaviours (note that B component is very weak) in the three stages. In fact, the trion is less intense in the less p-doped situation that corresponds to the vacuum annealing. The parallel shift of the energies of the three components is consistent with the lowering of the Fermi level in the conduction band as hole doping raises and the enhanced intensity with hole doping seems to be dominated by the healing of sulphur vacancies by O_2_.

In the present samples, the very disperse behaviour of the PL intensity with size (Figure 7b) is due to the combined occurrence of two opposite factors: on one hand larger p-doping is related to higher intensity but the formation of defects (revealed by the increased FWHM and Δ*ω*) depletes the PL. The higher p-doping as size is reduced can be related to the higher density of sulphur vacancies at the edges of the flakes that facilitate oxygen adsorption. Edges have a progressively more important weight as the size of the flakes shrinks.

## 4. Conclusions

Single- and few-layer (2–3) MoS_2_ nanoflakes have been obtained by CVD (10–500 nm) and PVT (1–5 μm) on sapphire substrates. The synthesis method does not have an important role in the behaviour of the flakes as their size is reduced except that the smallest are only obtained by CVD. The resulting flux of the precursors and the time allowable to react on the substrate, dictated by the Ar flux and the pressure, seem to be the relevant parameters that control the size of the flakes.

The standard relation in 2D-MoS_2_ between number of layers and frequency difference, Δ*ω*, is not adequate for nanoflakes since doping and strain modify the frequencies invalidating the correlation. The large change in the C exciton energy, obtained from reflectance or transmittance spectra, allows differentiating single-layer from few-layer flakes. It is then possible to analyse in detail the characteristics of the Raman phonons and PL band for the 1L and few-layer samples independently. We have used the Raman frequency difference, Δ*ω*, and the peaks widths as indicators of the presence of defects and disorder in the nanoflakes. No correlation is found between size and defects concentration, however, the few-layer nanoflakes do have, in general, higher disorder. Single-layers down to the smallest sizes (10 nm) present the characteristic Raman spectra of high quality MoS_2_. Moreover, as size shrinks, the strain imposed by the substrate is reduced so that, for 10 nm, the E_2g_ frequency coincides with relaxed large pristine flakes.

The observed increasing hole-doping as the flake size is reduced can be due to the preferential formation of S vacancies at the edges of the flakes and the healing of these defects by O_2_ adsorption. The edge/surface ratio increases as size is reduced, so the hole concentration is higher. Increased hole doping is evidenced by the A_1g_ phonon hardening and the blue-shift of the PL band. A study of the impact of annealing in air (oxygen rich) and vacuum (oxygen poor) atmospheres shows the relevance of O_2_ adsorption, consistent with the proposed mechanism for the p-doping. The single-layer nanoflakes maintain a PL efficiency similar or even higher than that of pristine large-area MoS_2_, thus the nano-flakes may be of interest for fabricating optical nano-devices for nano-photonics.

## Data Availability

Data presented in this article are available at request from the corresponding author.

## Supplementary Materials

The following supporting information can be downloaded at: https://www.mdpi.com/article/10.3390/nano12081330/s1. Figure S1: E_1g_ mode in Raman spectra of 1L and 2L MoS_2_, Figure S2: Fits of the PL bands versus air and vacuum annealing.
