# Supplementary material for: Size Effects in Single- and Few-Layer MoS2 Nanoflakes: Impact on Raman Phonons and Photoluminescence
Source: Nanomaterials (Basel). 2022 Apr 12;12(8):1330. doi: 10.3390/nano12081330 (PMC9027366; doi:10.3390/nano12081330)
Supplement: Supplementary file 1 [file nanomaterials-12-01330-s001.zip › nanomaterials-1613105-supplementary.pdf]

Supplementary Materials

Size effects in single- and few-layer MoS_2 nanoflakes: impact on the photoluminescence

Sandra Cortijo-Campos, Carlos Prieto and Alicia de Andrés*

Instituto de Ciencia de Materiales de Madrid, CSIC, C/ Sor Juana Inés de la Cruz, Cantoblanco 28049 Madrid, Spain

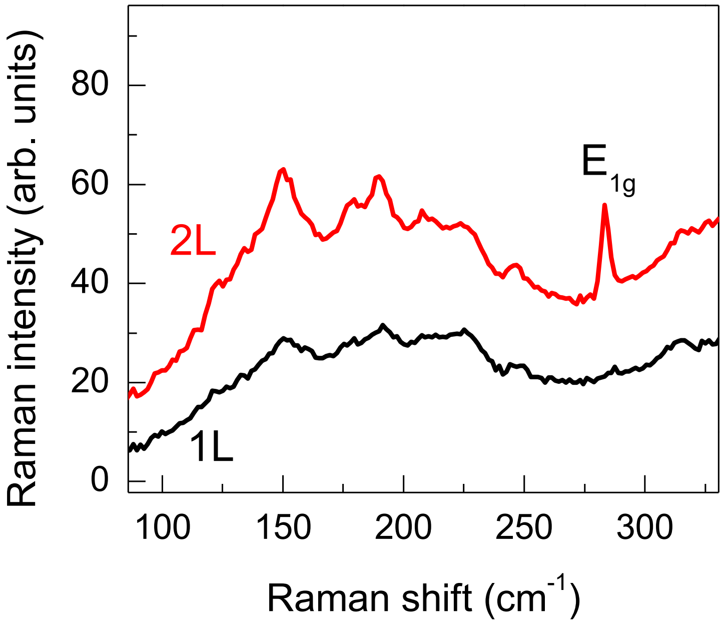

Figure S1. Raman spectra of 1L and 2L large MoS_2 flakes showing the emergence of the forbidden E_{1g} peak in the 2L flake.

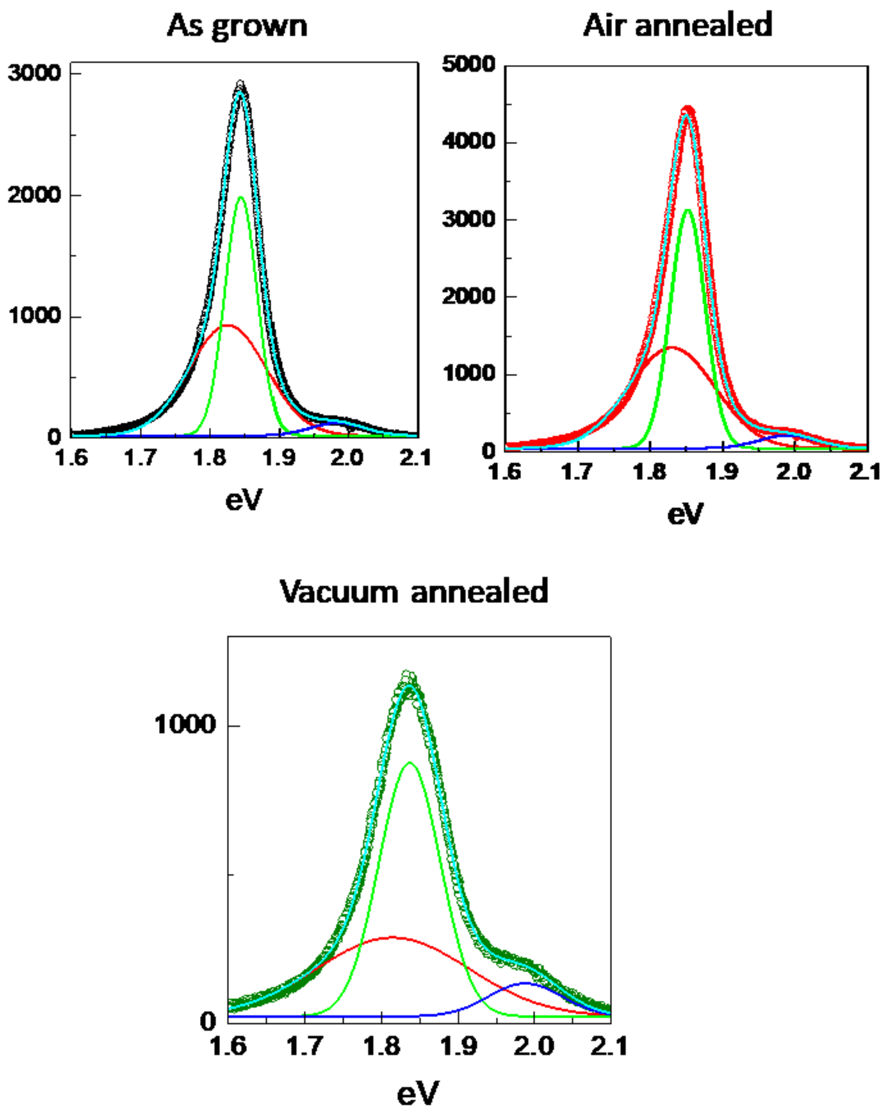

Figure S2. Fits (continuous cian lines) to the PL spectra (open symbols) of single-layer MoS_2 samples: asgrown, after an annealing in air and after a second annealing in vacuum. Red lines: negative trion X^-, green: A exciton, blue: B exciton.
